# FOXO3-induced microRNA-128-3p promotes the progression of spinal cord injury in mice via regulating NLRP3 inflammasome-mediated pyroptosis

**DOI:** 10.3389/fimmu.2025.1526721

**Published:** 2025-02-21

**Authors:** Shuo Yang, Yunzhi Guan, Chaojun Zheng, Xinlei Xia, Xiaosheng Ma, Jianyuan Jiang

**Affiliations:** Department of Orthopedics, Huashan Hospital, Fudan University, Shanghai, China

**Keywords:** MiR-128-3p, NLRP3-mediated pyroptosis, ROS, neuroinflammation, spinal cord injury

## Abstract

**Background:**

Spinal cord injury (SCI) remains a severe condition with an extremely high disability rate and complex pathophysiologic mechanisms. Pyroptosis, an inflammatory form of cell death triggered by certain inflammasomes, has a key role in a variety of inflammatory diseases, including SCI. However, it is unclear whether microRNAs (miRNAs), novel regulators in the SCI, are involved in SCI-induced pyroptosis.

**Methods:**

Two GEO miRNA expression profiles (GSE158195 and GSE90452) were downloaded, and the differentially expressed miRNAs were analyzed by bioinformatics methods. An *in vivo* animal model and an *in vitro* cellular model of SCI were constructed in female C57BL/6 mice and BV-2 cells for studying the possible roles of FOXO3, miR-128-3p and NLRP3-mediated pyroptosis in SCI. Markers of ROS, cell pyroptosis and inflammation were measured by RT-qPCR, Western blotting, immunofluorescence, flow cytometry, and enzyme-linked immunosorbent assays. Histopathological changes in spinal cord tissue were detected using hematoxylin and eosin and immunohistochemical. The Basso–Beattie–Bresnahan (BBB) score was used to evaluate the motor function of mice in each group.

**Results:**

Bioinformatics analysis of GSE158195 and GSE90452 datasets revealed a significant downregulation of miR-128-3p, a phenomenon that was consistently observed in the SCI mice model. Functionally, miR-128-3p upregulation improved functional behavioral recovery, relieved pathological injury, repressed oxidative stress, and alleviated pyroptosis and inflammation in the mouse SCI models. We also confirmed that Thioredoxin-interacting protein (TXNIP) was the target gene of miR-128-3p, and overexpression of TXNIP can effectively reverse the improvement of miR-128-3p in SCI cell model. Moreover, we found that transcription factor FOXO3 facilitated miR-128-3p expression, and its overexpression resulted in similar effects of miR-128-3p in the SCI cell model.

**Conclusion:**

To the best of our knowledge, this is the first report demonstrating miR-128-3p improved secondary injury in SCI through the modulation of cell pyroptosis pathway. Our results suggest that FOXO3/miR-128-3p/TXNIP/NLRP3-mediated pyroptosis axis may be a potential therapeutic target for SCI.

## Introduction

Spinal cord injury (SCI) is a grave form of trauma that can result in enduring impairments of motor and sensory functions, profoundly impacting an individual’s quality of life and autonomy (1). The pathophysiological process of SCI is bifurcated into two stages: primary and secondary injury. The primary injury, instigated by the mechanical force applied on the spinal cord, after which irreversible damage of neural cells and spinal tissue ensues. Secondary injury closely follows the primary injury, which is characterized by a cascade of pathological changes, such as oxidative stress, inflammatory reactions, apoptosis, and other mechanisms (2, 3). The reversibility of certain pathological aspects of secondary injury opens up avenues for therapeutic intervention in SCI.

Oxidative stress is a key factor in SCI and plays a very important role in this disease. After the primary injury, the damage to the microcirculation leaves the adjacent tissues in a state of hypoperfusion, and the resulting ischemia and hypoxia further lead to the generation of a large number of free radicals, represented by reactive oxygen species (ROS) (4). Elevated ROS can cause cell membrane damage and organelle dysfunction via lipid peroxidation, which then leads to a cascade of secondary injury events, including disruption of calcium homeostasis and release of excitatory amino acids. All these events, in turn, may further lead to mitochondrial dysfunction and increased ROS production, resulting in a vicious cycle that ultimately leads to neural cell death (5, 6). Furthermore, substantial evidence indicates that ROS played a key role in stimulating the inflammatory reaction by activating the NOD-like receptor pyrin domain-containing protein 3 (NLRP3) inflammasome (7). It has been determined that NLRP3 activation-mediated pyroptosis is involved in the aggravation of SCI (8, 9). Moreover, the previous study reported that inhibiting the activation of NLRP3 not only downregulates pyroptosis, but also alleviates SCI (10, 11). Therefore, manipulating ROS/NLRP3 induced pyroptosis might be a promising strategy to prevent SCI.

Pyroptosis, a distinctive type of programmed cell death, has been discovered in recent years. Unlike other forms of cell death, pyroptosis is characterized by cell swelling, rupture, and release of cytoplasmic contents (12, 13). Pyroptosis is closely related to the occurrence of central nervous system (CNS) injuries such as traumatic brain injury, cerebral ischemia, and SCI (14). Mechanistically, following CNS injury, activated NLRP3 recruits ASC and procaspase-1 to form the NLRP3 inflammasome. In this context, procaspase-1 would be cleaved to active caspase-1 by the NLRP3 inflammasome, which would lead to the mature processing of the inflammatory cytokines IL-18, IL-1β and GSDMD. IL-18 and IL-1β aggravate inflammatory damage, while GSDMD causes cell membrane perforation and eventually leads to cell lysis and the generation of a nonbacterial inflammatory response to pyroptosis (15). In addition, extensive studies have demonstrated that a series of pathological changes such as hemorrhage, hypoxia, and edema after SCI can cause accumulations of ROS, which can act as secondary messengers to induce NLRP3 inflammasome-mediated pyroptosis (16, 17). Although recent studies demonstrate that NLRP3-mediated pyroptosis plays crucial roles in the pathogenesis of multiple neurological diseases including SCI, the precise mechanisms in SCI remain to be fully elucidated.

MicroRNA (miRNAs), a class of small non-coding RNA molecules, exerts regulatory control over gene expression by interacting with mRNAs, inhibiting their translation into proteins (18). In the context of SCI, the expression profiles of certain miRNAs are significantly altered, which influences a variety of post-injury processes, including the inflammatory response, cell apoptosis, and oxidative stress (19, 20). Increasing evidence has demonstrated that miRNAs exert a significant regulatory effect on pyroptosis in various forms of tissue injury including SCI (21). For example, over-infiltration of Treg cell in mice by tail vein injection promote functional recovery by inhibiting microglia pyroptosis *in vivo*, which is achieved through Treg cell-derived exosomes miR-709 (22). Liu et al. Showed that that iPSC-NSCs-derived exosomes can package and deliver let-7b-5p, regulating the expression of LRIG3 to ameliorate microglia/macrophage pyroptosis and enhance motor function in mice after SCI (23). Additionally, a study has demonstrated that miR-128-3p exhibited a significant decrease in expression within rat models of SCI, correlating with the neuropathic pain that accompanies such injuries (24). The anti-inflammatory and anti-apoptotic properties of miR-128-3p have been previously reported (25). Inhibiting miR-128-3p stimulated oxygen-glucose deprivation and re-oxygenation (OGD/R)-induced pyroptosis in SH-SY5Y cells, while upregulating miR-128-3p attenuated OGD/R injury (26). However, not much was known the role and mechanisms of miR-128-3p in the modulation of NLRP3-mediated pyroptosis in SCI.

The aim of this study was to reveal the role and mechanisms of miR-128-3p-regulated pyroptosis signaling axis in the development and progression of SCI. Firstly, the differentially expressed miRNAs were screened by bioinformatics methods from two GEO miRNA expression profiles (GSE158195 and GSE90452). Subsequently, the improvement of neurological dysfunction, post-SCI neuroinflammation and subsequent neuropathology by miR-128-3p were verified in mice SCI models. Then, the possible mechanisms of these effects were investigated in LPS induced BV-2 cell injury model. Our findings suggest that FOXO3/miR-128-3p/NLRP3-mediated pyroptosis axis may serve as a new therapeutic target for the prevention and treatment of SCI.

## Materials and methods

### miRNA microarray

Microarray datasets GSE158195 and GSE90452 were obtained from the National Center for Biotechnology Information (NCBI) GEO database (http://www.ncbi.nlm.nih.gov/geo) to identify SCI-associated miRNAs. GSE158195 and GSE90452 datasets were based on Illumina HiSeq 2500 platform. The Limma package (R language) was applied for intergroup difference analysis with |logFC|> log_2_1.5 and P < 0.05 as the cutoffs for GSE158195 and GSE90452, followed by heatmap plotting of differentially expressed genes using the R-pHEATMAP package.

### Cell culture

The murine BV2 microglial cell line was obtained from Shanghai Cell Research Center (Shanghai, China). The cells were cultured in DMEM (4.5 g/L glucose) containing 10% FBS and 1% penicillin/streptomycin at 37 C in a 5% CO_2_ atmosphere. When the cells reached to approximately 80% confluence, they were digested with trypsin and passaged for additional experiments. To generate ROS, apoptosis, and inflammation, BV2 cells were stimulated for 8 h at 37°C with 100 ng/ml of lipopolysaccharide (LPS; Sigma-Aldrich, St. Louis, MO, USA).

### Cell pyroptosis analysis

For cell pyroptosis analysis, the *in vitro* FAM-FLICA Caspase-1 Detection kit (ImmunoChemistry Technologies, LLC) was used according to the manufacturer’s instructions. Briefly, the cells were harvested and washed with PBS. Subsequently, the cells were stained with 2 µg/ml PI and 10 µl FAM-FLICA. In each analysis, 20,000 gated events were recorded. The fluorescence intensity was measured using a FACSCalibur II flow cytometer and CellQuest software, version 5.1 (BD Biosciences)

### Luciferase reporter assay

TargetScan (http://www.targetscan.org/vert_72/), and miRDB (http://mirdb.org/) were used to predict the targeting mRNAs of miR-128-3p. To confirm the TXNIP 3’-UTR as a target of miR-128-3p, a dual luciferase reporter assay was conducted. Briefly, the wild-type (WT) or mutant (MUT) 3’-UTR of TXNIP was sub-cloned into the pGL3 luciferase reporter vector. Cells were then co-transfected with the miR-128-3p mimics, miR-128-3p inhibitor, mimics NC or inhibitor NC (5 nM, GeneChem Corp.), along with 100 ng of either the pGL3-WT-TXNIP or pGL3-MUT-TXNIP 3′-UTR using Lipofectamine 2000 (Invitrogen; Thermo Fisher Scientific, Inc.). Using the Dual-Luciferase Reporter Assay System (Promega Corporation), luciferase activity was assessed 48 h after transfection. The luciferase activity was normalized to the Renilla luciferase activity.

For the miR-128-3p promoter activity assay, the genomic sequences near the miR-128-3p TSS were amplified and cloned into the pGL3-Basic reporter vector (Promega). BV2 cells were cotransfected with the pGL3-Basic derived reporter vectors and Renilla luciferase plasmid, pRL-TK, and pcDNA-FOXO3. The cells were lysed at 48 h after transfection, and luciferase activity was measured using the Dual-Luciferase Reporter Assay System on a GloMax 96 Microplate Luminometer (Promega). The activities of firefly luciferase (PGL3-Basic) were normalized to Renilla luciferase (pRL-TK) activities.

### Western blot assay

Briefly, proteins were extracted by RIPA buffer (Vazyme Biotech Co., Ltd.) and quantitated with a bicinchoninic acid (BCA) assay kit (Thermo Fisher Scientific, Inc.). Subsequently, 30 µg proteins were loaded on 10% SDS-PAGE, transferred to PVDF membranes, and then blocked with 5% non-fat milk for 1 h at room temperature. Then, membranes were incubated with the primary antibodies: rabbit anti-Pro-Caspase-1 (1:1000 Abcam, UK), rabbit anti-Caspase-1 (1:2000 Affinity Biosciences, USA), rabbit anti-NLRP3 (1:1000 Abcam, UK), rabbit anti-GSDMD (1:1000 Bioworld, USA), rabbit anti- ASC (1:1500 Affinity Biosciences, USA) and rabbit anti-β-Actin (1:1000 Cell Signaling, USA) at 4°C overnight, followed by incubation with secondary horseradish peroxidase (HRP)-linked secondary anti-rabbit IgG antibody (1:10,000; Cell Signaling Technology, Danvers, MA, USA) at room temperature for 2 h. The films were scanned using the Pierce ECL Western Blotting Substrate (Pierce; Thermo Fisher Scientific, Inc.). The proteins were quantified using Quantity One software, version 4.2.1 (Bio-Rad Laboratories, Inc.).

### Chromatin immunoprecipitation assays

ChIP assays were performed using the EZ-Magna ChIP One-Day Chromatin Immunoprecipitation Kit (Millipore, USA) according to the instruction manual. Briefly, approximately 1x10^7^ BV2 cells were cross-linked and lysed by lysis buffer containing Protease Inhibitor Cocktail II. Chromatin was sheared by sonication (high power, 10 cycles of 30 s ‘on’ and 30 s ‘off’; Bioruptor UCD-300, USA) to an average size of 200 to 1000 bp, and the sheared chromatin samples were divided into 50-μl aliquots. Each aliquot of chromatin (1x10^6^ cell equivalents of chromatin) was diluted in 450 μl of dilution buffer. Five microliters (1%) of the supernatant were removed as ‘Input’, and the immunoprecipitating antibody, FOXO3 (Abcam, ab26109, 5 μg, UK), and 20 μl of protein A magnetic beads were added to the remaining supernatant. Finally, the immune complexes were collected, washed, and eluted, and the cross-links were then reversed. DNA was recovered and analyzed by qRT-PCR using specific primers. As a negative control, normal mouse IgG was used for immunoprecipitation.

### Animals

Adult female C57BL/6 mice, weighing 20-25 g, specific pathogen-free (SPF) grade were provided by SLAC Laboratory Animal Co., Ltd. in Shanghai, China. All operations involving animal care and experimentation were evaluated and approved by the Experimental Animal Ethics Committee of Huashan Hospital, Fudan University, under approval number 20230149. All mice were housed in a clean-grade animal room with a 12-h light/dark cycle, room temperature of 25°C with free access to food and water.

### Experimental design

Mice were randomly divided into three groups to determine the role of miR-128-3p: Injury group, Injury + agomir-miR-128-3p, and Injury + agomir-NC. In Injury + agomir-miR-128-3p/agomir-NC group, the mice were injected with agomir-miR-128-3p/agomir-NC (20 µM, 2 µL) using a glass micropipette (tipdiameter 20–40 µm) via intrathecal injection according to previous studies (3, 27). 28 days following the surgery, the mice were humanely killed with i.p. injection of 50 mg/kg pentobarbital sodium followed by cervical dislocation, and subsequently, the injury spinal cord from the injury center were isolated for reverse transcription-quantitative polymerase chain reaction (RT-qPCR), HE and Cresyl violet staining, as well as Western Blot.

### SCI surgery

To establish SCI animal models, mice are first anesthetized with pentobarbital (Sigma-Aldrich, St. Louis, MO, U.S.A.) at a dosage of 50 mg/kg via intraperitoneal (i.p.) injection. Following anesthesia, a laminectomy is performed at the T9-T10 vertebral level to expose the underlying spinal cord while keeping the dura intact. Next, the spinous processes of T8 and T11 are clamped to stabilize the spinal column. The dorsal surface of the exposed spinal cord is then subjected to a weight drop injury inflicted by a New York University impactor (New York, NY, USA), using a 10 g weight dropped from a height of 25 mm. The success of the SCI model is determined by two key indicators: the presence of a complete clamp trace and the onset of paralysis in both lower limbs post-awakening. Any model that does not exhibit these signs is not considered successful and is excluded from further experimental procedures. Sham-operated mice were treated the same as the SCI-treated mice except for no contusion.

### Functional analysis

Neural function was assessed at 1, 7, 14, 21, and 28 days after SCI by 21-point BBB scores. The scale went from 0 (completely paralyzed) to 21 (normal) (28). During the assessment, three observers who did not know the grouping situation analyzed the hindlimb locomotor activity, stability, coordination, stepping, trunk position, the gap, foot position, and tail position of the mice, respectively, to obtain the average.

### Tissue preparation

For total protein extraction and qRT-PCR assay, the injured spinal cord segments were directly separated after euthanasia and then immediately preserved in liquid nitrogen. For paraffin sectioning, the spinal cord tissue was embedded in paraffin after post-fixing overnight. Transverse sections (10 μm) were taken for Hematoxylin-Eosin (HE) staining and Cresyl Violet Staining. For spinal cord water content measurement, with the spinal cord injury site as the center, 0.5 cm was taken from the cephalic and caudal sides (29). According to the grouping of experimental animals, 6 samples were collected separately for each group.

### HE and cresyl violet staining

A 10 mm long segment of the spinal cord containing the epicenter from indicated groups was fixed with 4% paraformaldehyde (Solarbio, China) for 30 min at room temperature, embedded in paraffin, and cut into 10-μm thick serial sections. One set of tissue sections was used for routine HE staining (Beyotime, China). The sections were stained in HE staining solution (10 min), and differentiated with 1% hydrochloric acid alcohol, treated with 2% sodium bicarbonate for blue returning (10 s), and stained with 0.5% eosin solution for 3 min at 25°C, followed by gradient alcohol dehydration, xylene clearance, and then neutral resin sealing. Finally, the tissues were dried for 72 h, and the whole sections were observed under a low magnification field (×50). The others were put onto Superfrost Plus Slides and every 40th section was stained with 0.5% cresyl-violet acetate and imaged using a BX51 light microscope (Olympus Inc., Tokyo, Japan). Using Image-Pro Plus 6.0 (Media Cybernetics, USA) software, the lesion area and spared tissue area were outlined and quantified as described previously [24].

### Spinal cord water content measurement

The spinal cord tissues obtained in the above experimental procedure at 7 days post-injury were immediately weighed, and then the dry weight of spinal cord tissues was obtained at 100°C for 24 h. The ratio of wet-to-dry weight is calculated as follows: [(wet weight − dry weight)/wet weight] × 100%.

### RNA isolation and quantitative RT-PCR

Total RNA was extracted from spinal cord tissues and cells with a miRNeasy Mini kit (Qiagen GmbH, Hilden, Germany). Reverse transcription of miR-128-3p was synthesized using the miScript II RT kit (Invitrogen, Carlsbad, CA). miR-128-3p expression was measured using the Exiqon SYBR Green Master Mix (Exiqon, Vedbaek, Denmark) on an ABI Prism 7500 HT (Applied Biosystems). The primers used for were as follows: miR-128-3p forward: 5′-GACTATCACAGTGAACCGGT-3′, reverse: 5′-AGTGCAGGGTCCGAGGTATT-3′; U6 forward primer: 5’-GCTTCGGCAGCACATATACTAAAAT-3′; U6 reverse primer: 5′-CGCTTCACGAATTTGCGTGTCAT-3′. The relative expression of each gene was calculated using the 2^−ΔΔCt^ method (30).

### Biochemical analysis

Spinal cord tissue was dissected and placed in pre-cooled PBS buffer and then homogenized with a homogenizer. The supernatant was collected by centrifugation at 3000 r/min for 20 min at 4 °C. For cultured cells, the supernatant was collected after treatment by centrifugation at 12000×g for 10 min. The activities of MDA, SOD, and GPX were determined by commercial kits according to the manufacturer’s instructions. TNF-α, IL-1β, and IL-18 were measured by ELISA kit (all from Beyotime, Shanghai, China) according to manufacturers.

The reactive oxygen species (ROS) activity in spinal cord tissue and BV-2 cells was determined by fluorescent probe dichlorodihydrofluorescein diacetate (DCFH-DA) assay using a ROS detection kit (Beyotime, Shanghai, China) by the instructions of the manufacturer.

### Immunohistochemical

The spinal cord segments were paraffin-embedded and cut into 5-µm-thick slides. Next, the tissue sections were deparaffinized and dehydrated through graded alcohols. The slices were then incubated in 3% H_2_O_2_ for 15 min at room temperature (RT) and then blocked in 10% bovine serum for 30 min. Subsequently, the slices were stained overnight at 4°C with a primary antibody against TXNIP (cat. no. 14715, Cell Signaling Technology, Inc, 1:200). Subsequently, the sections were incubated with secondary antibodies (cat. no. 7074; Cell Signaling Technology, Inc. 1:2,00). Finally, the immunoreactivity was visualized by staining with diaminobenzidine (DAB) for 3 min, covered with a coverslip, and analyzed under a light microscope (BX51, Olympus Inc., Tokyo, Japan) at 200× magnification.

### Statistical analysis

Data were expressed as mean ± SD. Statistical analysis was conducted by SPSS software (SPSS Inc., Chicago, IL, USA) through *post hoc* multiple comparisons of one-way analysis of variance (ANOVA). If an ANOVA F-value was significant, then *post hoc* comparisons were performed among groups. Differences were considered significant when p < 0.05.

## Results

### MiR-128-3p was downregulated in the spinal cord tissues of mice after SCI

**Figure 1:**
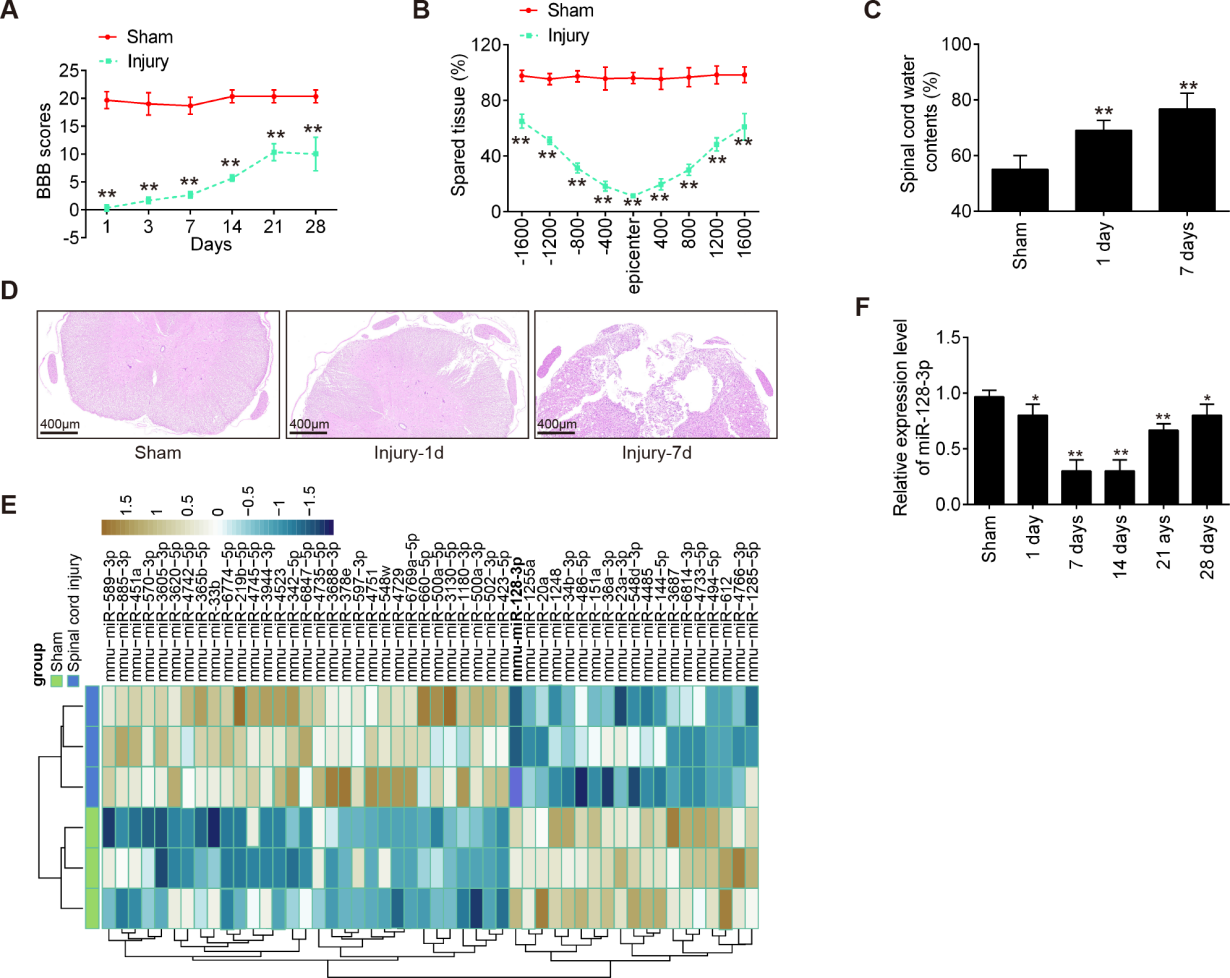
MiR-128-3p is downregulated in the spinal cord tissue of SCI mice. **(A)** The BBB scores at 1, 3, 7, 14, 21, and 28 days after SCI were shown for all groups of mice (n = 6/group). **(B)** Quantification of spared tissue within the injury site, and 1600 μm rostral and caudal to the epicenter, 7 days post injury (n = 6/group). **(C)** Spinal cord water content was assessed using wet-to-dry weight method (n = 6/group). **(D)** The staining images of spinal cord tissues following Hematoxylin and eosin (HE) staining under a low magnification field (×50) (n = 6/group). **(E)** A cluster heap map was used to present the upregulated and downregulated miRNAs from GSE158195 and GSE90452. **(F)** qRT-PCR was performed to detect the expression of miR-128-3p in spinal cord tissues from mice at 1, 7, 14, 21, and 28 days after SCI (n = 6/group/time). Data represent the mean ± SD of three independent experiments. *p < 0.05, **p < 0.01 *vs*. Sham group.

First, we established an SCI mice model as above described, and then the behavioral analyses of the mice were evaluated. As shown in **Figure 1A**, BBB scores were significantly lower in the Injury group than that in the Sham group. Cresyl Violet staining was used to assess spared tissue following behavioral analyses. Sections of the thoracic spinal cord were analyzed and the ratio for “injured area/total area” from each section was determined. The spared tissue was smaller in SCI mice not only at the injury epicenter but also in regions extending away from the epicenter, in both rostral and caudal directions, compared with the Sham group (**Figure 1B**). Spinal cord edema was evaluated by detecting the water content of the spinal cord. It was observed that water contents in the spinal cord were significantly increased compared with the Sham group and was maximal at 7 days (**Figure 1C**). Moreover, the spinal tissues of mice were sliced and stained with H&E. Staining results demonstrated severe damage to the blood-spinal cord barrier and the structural integrity of the lesion epicenter, including rupture, hemorrhage and inflammatory cell infiltration (**Figure 1D**). All data indicated that the SCI model was successfully constructed.

To further determine whether miRNAs were involved in the SCI, we downloaded and analyzed the GSE158195 and GSE90452 datasets from the GEO database. Differentially expressed miRs were selected under the screening criteria |log2(Fold Change)| > 1.5) and P < 0.05. The dysregulated miRNAs are shown in **Figure 1E**. According to the data, we summarized the dysregulated miRNAs in the SCI samples compared with those in the sham samples, as follows: 31 miRNAs were upregulated, and 19 miRNAs were downregulated. Among them, miR-128-3p exhibited the most down-regulated alteration in the spinal tissues of SCI mice. Previous studies have shown that down-regulation of miR-128 contribute to the development of neuropathic pain (NPP) following SCI via activation of P38 (24). However, whether miR-128-3p can exert protective effects against inflammatory response and apoptosis in secondary SCI remains unclear. Therefore, we chose miR-128-3p for further investigation.

Next, the expression changes of miR-128-3p were calculated in the spinal cord tissues of mice at different time points. As shown in **Figure 1F**, the miR-128-3p expression levels were reduced at different time points after SCI compared with the expression in the sham group. The decrease in the level of expression of miR-128-3p was lower than Sham group, especially at 7 and 14 days after injury. All data indicates that miR-128-3p may be involved in the pathogenesis of SCI.

### Agomir-miR-128-3p injection improved functional recovery of SCI mice

**Figure 2:**
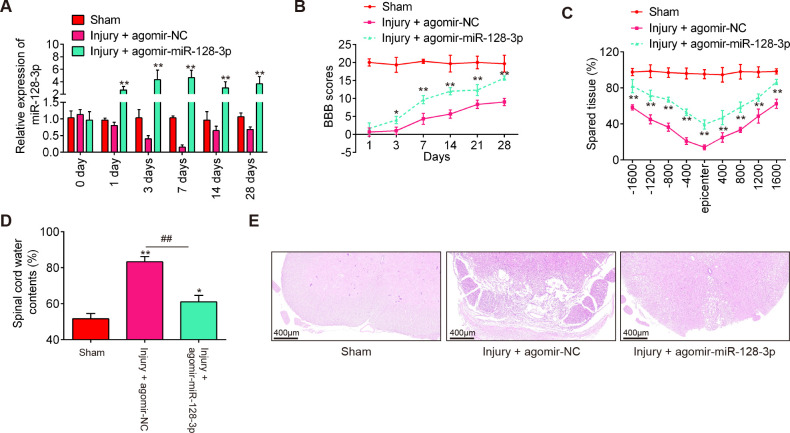
Agomir-miR-128-3p improves recovery of SCI mice. The mice were subjected to SCI and treated intrathecally with agomir-miR-128-3p/agomir-NC. At indicated time, the animals were sacrificed following pentobarbital sodium (50 mg/kg, i.p) anesthesia, and subsequently, a 10 mm long segment of the spinal cord was harvested for further experiments. **(A)** qRT-PCR was performed to determine the expression levels of miR-128-3p in spinal cord tissues at 1, 3, 7, 14, and 28 days after agomir-128-3p injection (n = 6/group/time). **(B)** The BBB scores at 1, 3, 7, 14, 21, and 28 days after SCI were shown for all groups of mice (n = 6/group). **(C)** Quantification of spared tissue within the injury site, and 1600 μm rostral and caudal to the epicenter, 7 days post injury (n = 6/group). **(D)** Spinal cord water content was assessed using wet-to-dry weight method (n = 6/group). **(E)** The staining images of spinal cord tissues following HE staining under a low magnification field (×50) (n = 6/group). Data represent the mean ± SD of three independent experiments. *p < 0.05, **p < 0.01 *vs*. Sham group; ##p < 0.01 *vs*. SCI + agomir-NC group.

To examine the impact of miR-128-3p in SCI, agomir-miR-128-3p and agomiR-NC were injected into SCI mice via intrathecal injection. miR-128-3p expression level was significantly increased in the spinal cord tissues of SCI mice from 1 to 28 days post-injury, compared with the agomiR-NC group (**Figure 2A**). Subsequently, the hindlimb motor function recovery of SCI mice was evaluated using the BBB score. Agomir-miR-128-3p injection indeed improved functional recovery from 3 days compared with that in the agomir-NC injection group (**Figure 2B**). Using Cresyl Violet staining assay, it was found that the amount of spared tissue was markedly increased in the Injury + agomir-miR-128-3p group compared with the agomir-NC group, indicating that agomir-miR-128-3p can reduce lesion size in SCI mice (**Figure 2C**). Meanwhile, the water contents in the spinal cord were found to be dramatically decreased after administering agomir-miR-128-3p, compared with the agomir-NC group (**Figure 2D**), suggesting the alleviation of spinal cord edema. As shown in **Figure 2E**, the injury group exhibited numerous red blood cells and inflammatory cells, especially glial cell proliferation and satellitosis, at the injury site. The spinal cord structure was disrupted, with blurred boundaries between gray and white matter and a significant reduction in neuron count. However, treatment with agomir-miR-128-3p significantly reduced the injury effects. The spinal cord structure was more intact, and the neuron count was higher compared to the injury group. Taken together, miR-128-3p up-regulation could promote functional recovery in SCI mice.

### Agomir-miR-128-3p injection suppressed the oxidative stress, cell pyroptosis and inflammation in SCI mice

**Figure 3:**
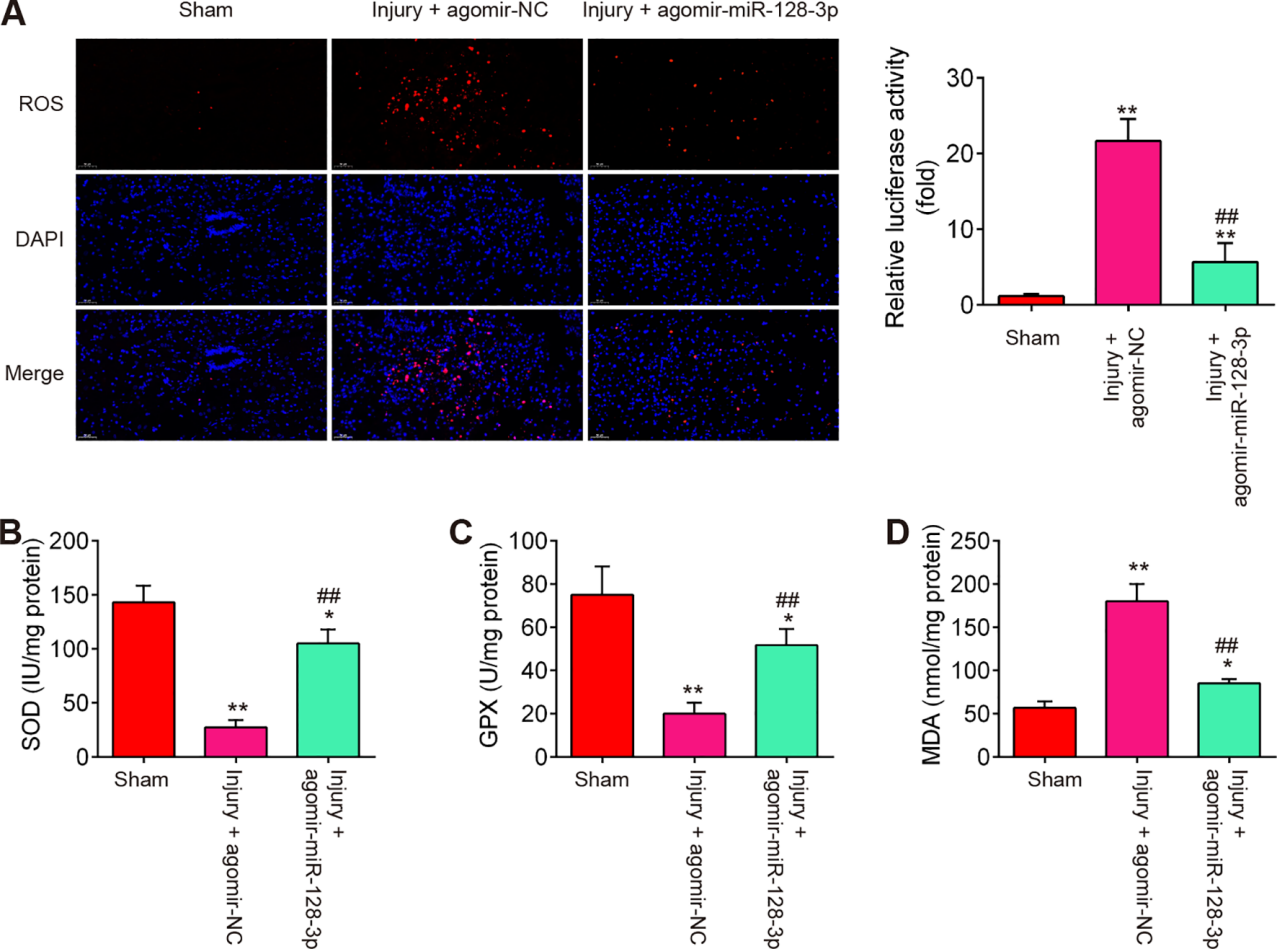
Agomir-miR-128-3p suppresses the oxidative stress of SCI mice. **(A)** ROS level in spinal cord tissues were detected by DCFH-DA probe. **(B–D)** The contents of SOD, GPX and MDA were measured by commercial kits. Data represent the mean ± SD of three independent experiments. *p < 0.05, **p < 0.01 *vs*. Sham group; ##p < 0.01 *vs*. SCI + agomir-NC group.

During SCI, excessive oxidative stress in neurons is a major contributor to the development of secondary injury (31), therefore, we sought to assess the effect of agomir-miR-128-3p on the oxidative stress in SCI mice. As shown in **Figures 3A–D**, the levels of ROS, the activity of SOD, and GPX were significantly increased, while the activity of MDA was markedly decreased in the Injury group compared with the Sham group. However, SCI-induced oxidative damage was alleviated by Agomir-miR-128-3p treatment.

**Figure 4:**
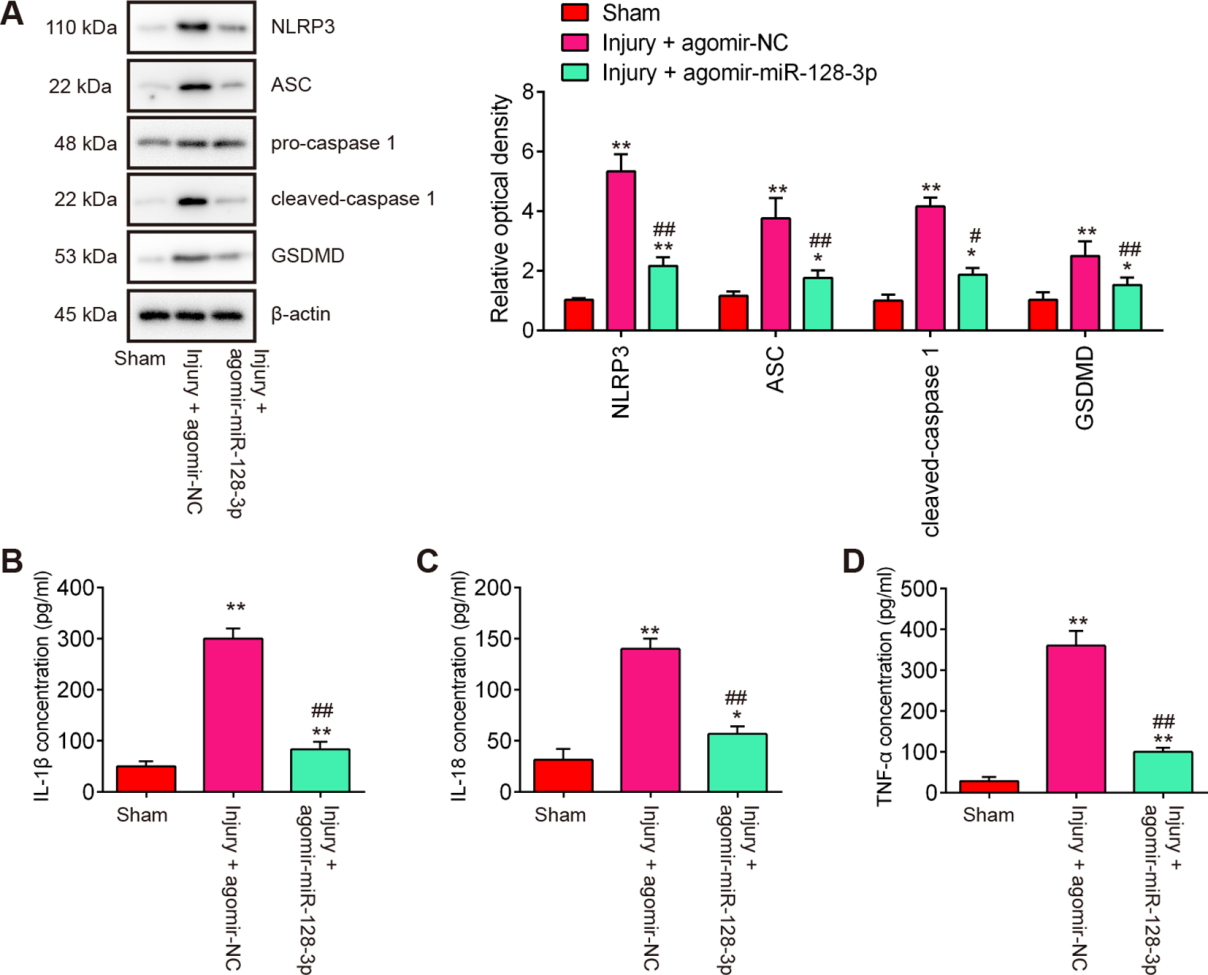
Agomir-miR-128-3p inhibits the cell pyroptosis and inflammatory response in SCI mice. **(A)** The expression levels of the NLRP3, ASC, Pro-Caspase-1, cleaved Caspase-1, and GSDMD proteins were measured by Western Blot. β-actin was used as a loading control. **(B–D)** The expression levels of IL-1β, IL-18 and TNF-α in spinal cord tissue were detected by ELISA. Data represent the mean ± SD of three independent experiments. *p<0.05, **p<0.01 *vs*. Sham group; #p<0.05, ##p<0.01 *vs*. Injury+ agomir-NC group.

During secondary injury, cell pyroptosis and pyroptosis-induced inflammation in neurons are other major pathogenic processes (22), therefore, we sought to assess the impact of agomir-miR-128-3p on the cell pyroptosis and inflammation in SCI mice. As shown in **Figure 4A**, the expression levels of pyroptosis-related genes including NLRP3, ASC, pro-Caspase-1, and GSDMD were significantly increased expressed in spinal cord tissues after SCI, compared with the Sham group, while they were decreased in the agomir-miR-128-3p group compared with the SCI group. Meanwhile, the pro-inflammatory mediators (IL-1β, IL-18, and TNF-α) in spinal cord tissues were also highly expressed, but decreased after treatment with the agomir-miR-128-3p in SCI mice (**Figures 4B–D**). All these data suggest that miR-128-3p may improve SCI through the regulation of microglia pyroptosis.

### TXNIP was a direct target of miR-128-3p

**Figure 5:**
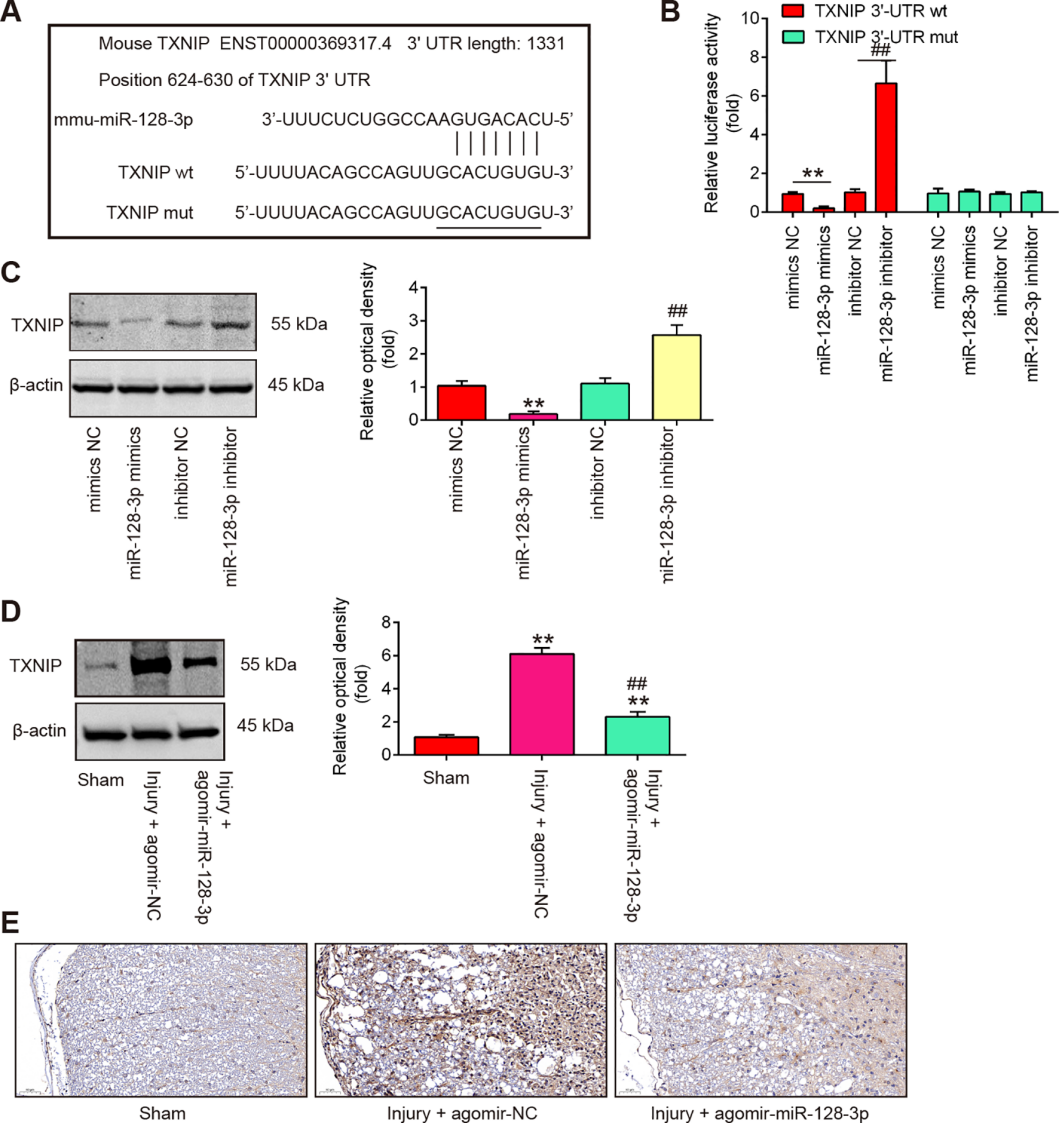
TXNIP is a direct target of miR-128-3p. **(A)** miR-128-3p-binding sequences and altered locations in the 3’-UTR of TXNIP. **(B)** Following transfection with miR-128-3p mimics or inhibitor, the relative luciferase activity of TXNIP wild type (wt) or mutant (mut) 3′-UTR in BV-2 cells is shown (n = 3). **(C)** Western blot was used to identify the TXNIP protein in BV-2 cells following treatment with miR-128-3p mimics or inhibitor. Data represent the mean ± SD of three independent experiments. **p < 0.01 *vs*. mimcis-NC group; ##p < 0.01 *vs*. inhibitor-NC group. **(D)** After injecting agomir-miR-128-3p, the protein TXNIP was identified by Western Blot in the spinal cord tissues of SCI mice. Data represent the mean ± SD of three independent experiments. **p < 0.01 *vs*. Sham group; ##p <0.01 *vs*. Injury+ agomir-NC group. **(E)** IHC was used to gauge the TXNIP expression levels in spinal cord tissues of SCI mice.

To explore the potential mechanisms in which miR-128-3p protected mice against SCI-oxidative stress and cell pyroptosis, we performed TargetScan 7.0 and Miranda to predict the targets of miR-128-3p. Bioinformatics analysis indicated that TNXIP was a potential target of miR-128-3p (**Figure 5A**). Previous studies have reported that TNXIP plays an amplifying role in tissue pathology and inflammation, leading to secondary damage after the initial SCI (32, 33). Moreover, TNXIP has been found to influence the pathways that lead to pyroptosis by modulating the activity of the NLRP3 inflammasome and the production of inflammatory mediators (34, 35). Thus, we chose it for the next study. Next, a luciferase reporter assay was then performed in BV-2 cells to determine whether miR-128-3p directly targets TNXIP. As shown in **Figure 5B**, miR-128-3p overexpression significantly repressed the luciferase activity, whereas miR-128-3p downregulation increased the luciferase activity of the TNXIP-3′UTR wt reporter plasmid, but not that of the mutant reporter. Furthermore, the results of Western blot analysis showed that miR-128-3p overexpression notably reduced protein levels of TNXIP, while miR-128-3p downregulation had the opposite effect in BV-2 cells (**Figure 5C**). It was also observed that the protein levels of TNXIP were significantly up-regulated in spinal cord tissues after SCI, whereas this increase was impaired by Agomir-miR-128-3p (**Figure 5D**). In addition, the same results were observed in the IHC assay (**Figure 5E**). All these data suggest that miR-128-3p may regulate pyroptosis-induced secondary injury through targeting TNXIP.

### Overexpression of miR-128-3p improved LPS-induced oxidative stress by targeting TNXIP in the SCI cell model

**Figure 6:**
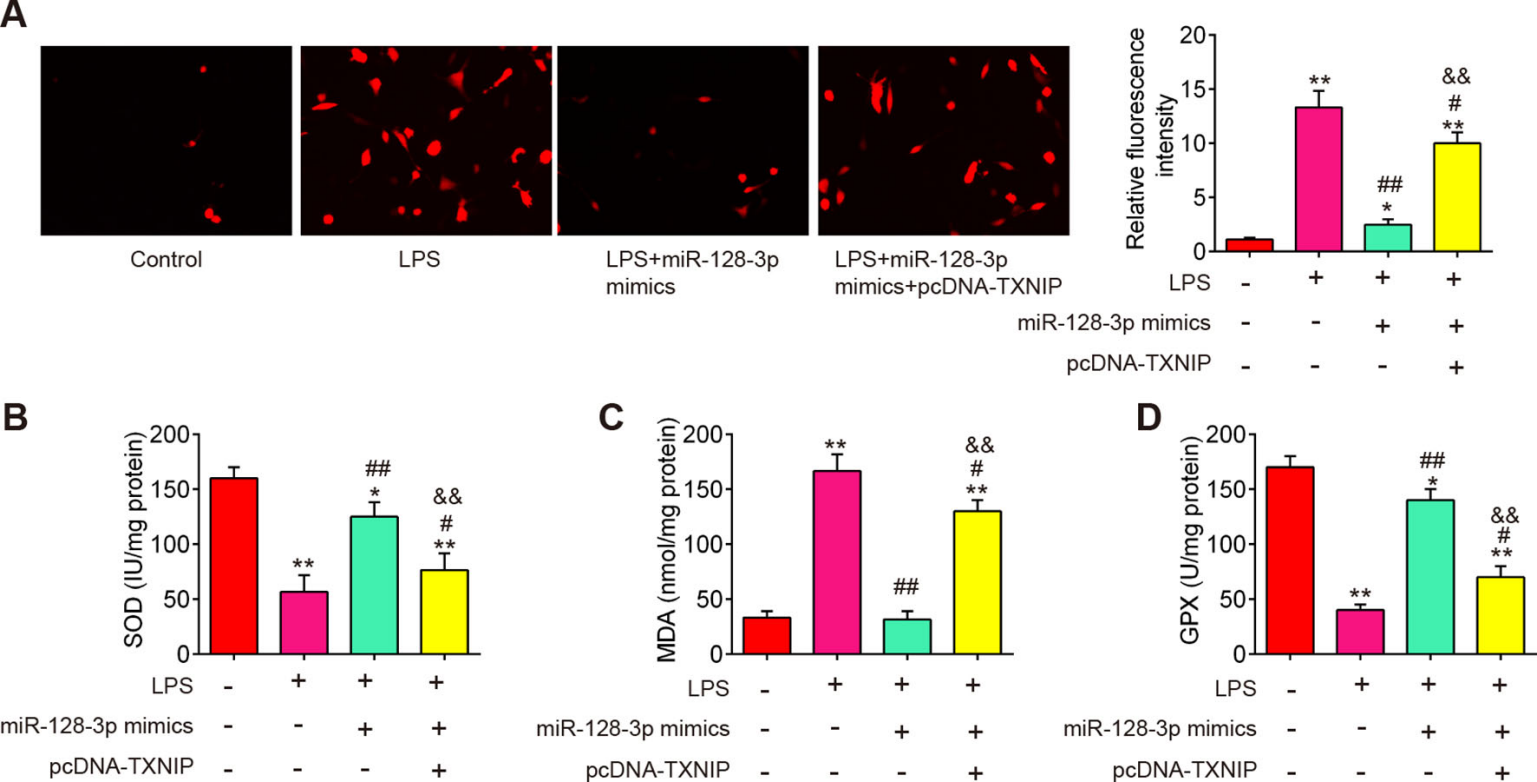
miR-128-3p inhibited ROS production by targeting TXNIP *in vitro*. miR-128-3p mimics were transfected into BV-2 cells (1 × 10^6^/well) in the presence of pcDNA-TXNIP plasmid, after 24 h, the cells were exposed to LPS (100ng/ml) for 8 h before being extracted for further research. **(A)** ROS levels as measured by DCF-DA Assay. **(B–D)** Commercial kits were used to measure the SOD, GPX, and MDA contents. Data represent the mean ± SD of three independent experiments. *p<0.05, **p<0.01 *vs*. Control group; #p<0.05, ##p<0.01 *vs*. LPS group. $$P<0.01 *vs*. LPS + miR-128-3p mimics group.

To explore the molecular mechanism involved in the protection of miR-128-3p in secondary SCI-induced oxidative stress, we established a SCI cell model using LPS-treated BV2 cells as previously described (36). To investigate the functions of miR-128-3p in LPS-induced BV-2 cell injury, the miR-128-3p mimics were transfected to BV-2 cells 4 h before LPS treatment. As shown in **Figures 6A–D**, the levels of ROS and MDA were significantly increased, while the activity of SOD and GPX was markedly decreased in LPS-treated BV-2 cells compared with the control group. However, LPS-induced oxidative damage was alleviated by miR-128-3p mimics treatment. Notably, the improvement of oxidative damage caused by miR-128-3p mimics was impaired by pcDNA-TNXIP. All these data suggest that miR-128-3p reduced SCI-induced oxidative stress by targeting TNXIP *in vitro*.

### Overexpression of miR-128-3p improved LPS-induced cell pyroptosis by targeting TNXIP in the SCI cell model

**Figure 7:**
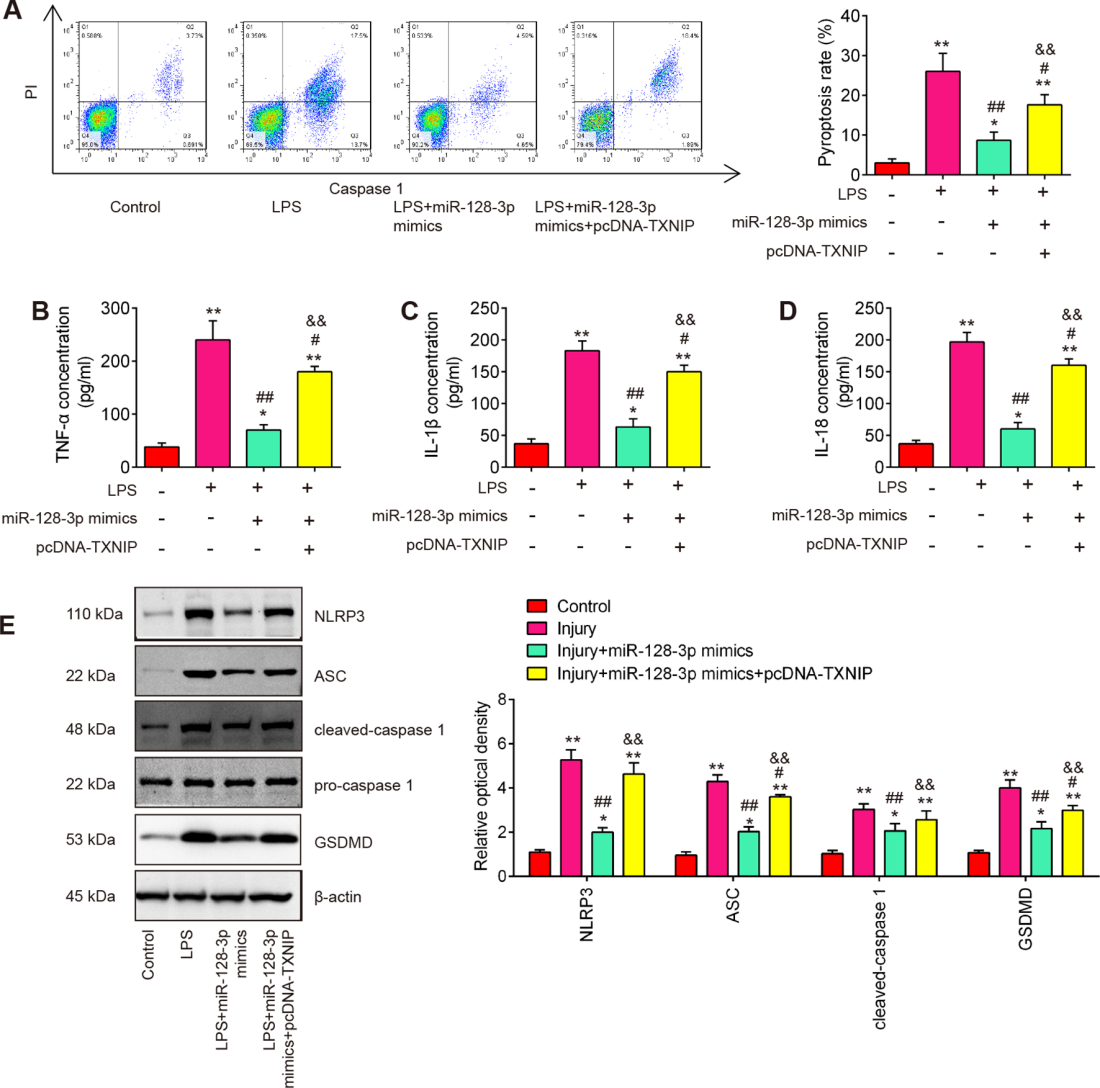
miR-128-3p inhibited cell pyroptosis and inflammation via regulating TXNIP expression *in vitro*. **(A)** Flow cytometry study to identify cell pyroptosis. **(B–D)** ELISA kits were used to find the levels of IL-1β, IL-18, and TNF-α expression in BV-2 cells. **(E)** Western blot analysis was used to determine the expression levels of the proteins NLRP3, ASC, Pro-Caspase-1, cleaved Caspase-1, and GSDMD. A loading control was performed with β-actin. Data represent the mean ± SD of three independent experiments. *p<0.05, **p<0.01 *vs*. Control group; #p<0.05, ##p<0.01 *vs*. LPS group. $$p<0.01 *vs*. LPS + miR-128-3p mimics group.

A previous study reported that TXNIP plays an important role in this process that ROS promotes the assembly and activation of the NLRP3 inflammasome (37). NLRP3 inflammasome is a multi-protein complex that plays a crucial role in the regulation of inflammation and cell death, particularly pyroptosis in various diseases (38). Therefore, we further explore whether miR-128-3p affects cell pyroptosis by targeting TNXIP in the SCI cell model. As shown in **Figure 7A**, the pyroptosis rate was significantly increased in LPS-treated BV2 cells, whereas the increased pyroptosis rate was markedly reduced by miR-128-3p mimics. Moreover, the improvement of miR-128-3p on the cell pyroptosis was reversed by pcDNA-TNXIP. In addition, the suppressive effect of miR-128-3p on LPS-induced inflammatory response (IL-1β, IL-18, and TNF-α) was also reversed by pcDNA-TNXIP (**Figures 7B–D**). Meanwhile, the increased expression levels of NLRP3, ASC, cleaved–Caspase-1 and GSDMD induced by LPS were significantly reduced by miR-128-3p mimics, whereas the inhibitory effects of miR-128-3p mimics were reversed by pcDNA-TNXIP (**Figure 7E**). All these data suggest that up-regulation of miR-128-3p suppressed LPS-induced cell pyroptosis and inflammation by targeting TNXIP.

### miR-128-3p expression is transcriptionally facilitated by FOXO3

**Figure 8:**
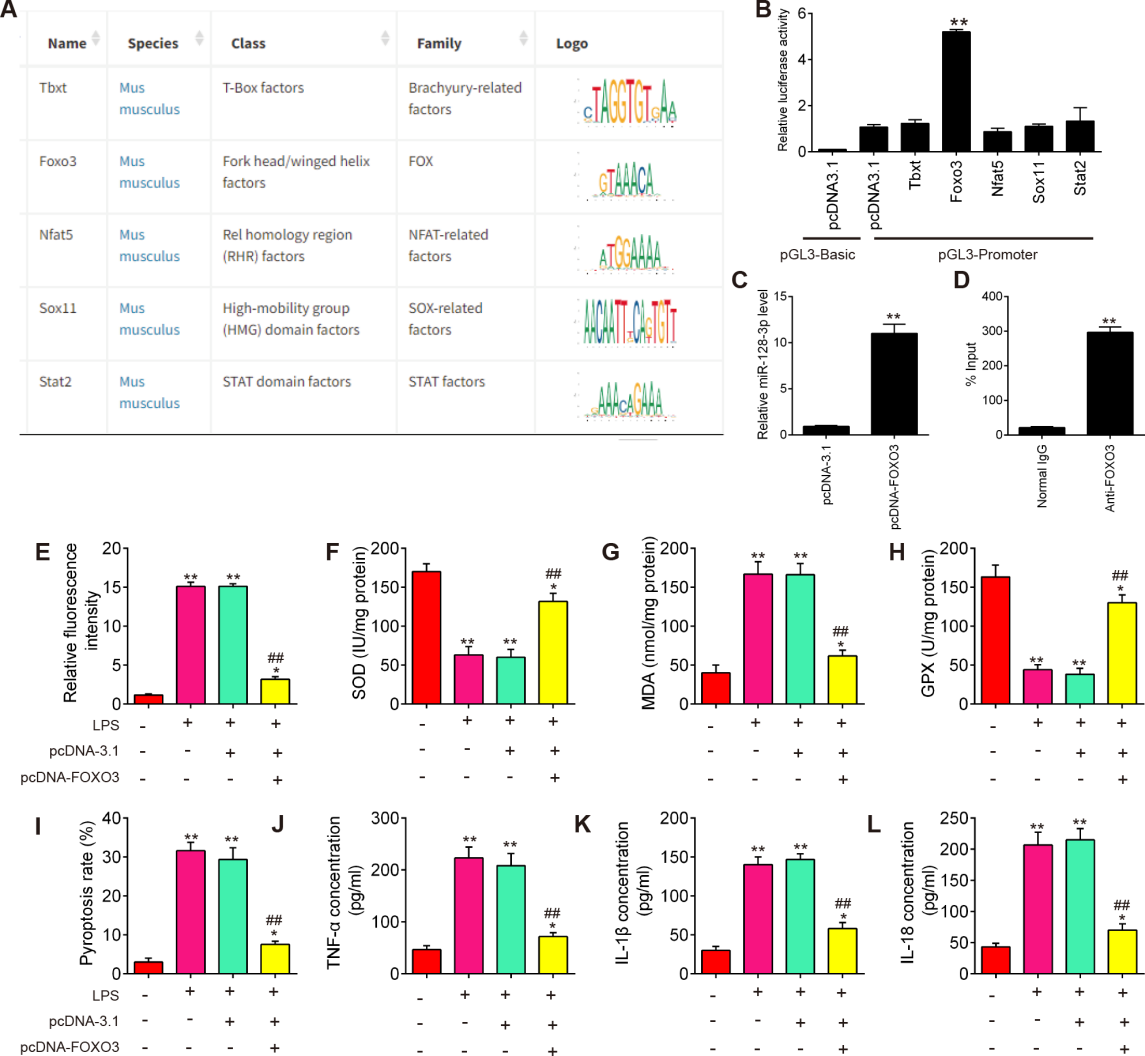
miR-128-3p expression is transcriptionally facilitated by FOXO3. **(A)** TRANSFAC soft was performed to predict promoter region of miR-138-3p. **(B)** Dual-luciferase assays show the effect of several transcription factors (Tbxt, FOXO3, Nfat5, Sox11, and Stat2) on miR-138-3p promoter activity. **(C)** qRT-PCR assays show the effects of FOXO3 overexpression on miR-138-3p expression. **(D)** Chromatin immunoprecipitation assays show the *in vivo* interaction between FOXO3 and the miR-128-3p promoter. Data represent the mean ± SD of three independent experiments. **p<0.01. **(E)** The effects of FOXO3 overexpression on ROS levels in SCI cell model. **(F–H)** The effects of FOXO3 overexpression on the SOD, GPX, and MDA contents in SCI cell model. **(I)** The effects of FOXO3 overexpression on cell pyroptosis in SCI cell model. **(J–L)** The effects of FOXO3 overexpression on the levels of IL-1β, IL-18, and TNF-α in SCI cell model. Data represent the mean ± SD of three independent experiments. *p<0.05, **p<0.01 *vs*. Control group; ##p<0.01 *vs*. LPS group.

To determine which transcription factors might influence miR-128-3p expression, we performed in silico analysis of the identified promoter region using TRANSFAC (http://www.biobase-international.com/product/transcription-factor-binding-sites) and several potential transcription factors (Tbxt, FOXO3, Nfat5, Sox11, and Stat2) were predicted (**Figure 8A**). To validate this prediction, we examined the effects of several potential transcription factors on miR-128-3p promoter-driven luciferase activity. Among these predicted transcription factors, overexpression of FOXO3 in BV2 cells significantly activated miR-128-3p promoter-driven luciferase activity (**Figure 8B**). Furthermore, the effect of FOXO3 overexpression on miR-128-3p expression was analyzed by qRT-PCR. As shown in **Figure 8C**, FOXO3 overexpression significantly increased endogenous mature miR-128-3p expression compared with the control vector, pcDNA3.1. These results indicate that miR-128-3p transcription is regulated by FOXO3. The interaction between FOXO3 and the miR-128-3p promoter was further confirmed by ChIP qRT-PCR assays. As shown in **Figure 8D**, the *in vivo* binding of FOXO3 to the miR-128-3p promoter was significantly higher than that of the control. Taken together, these findings suggest that miR-128-3p expression is transcriptionally facilitated by FOXO3.

**Figure 9:**
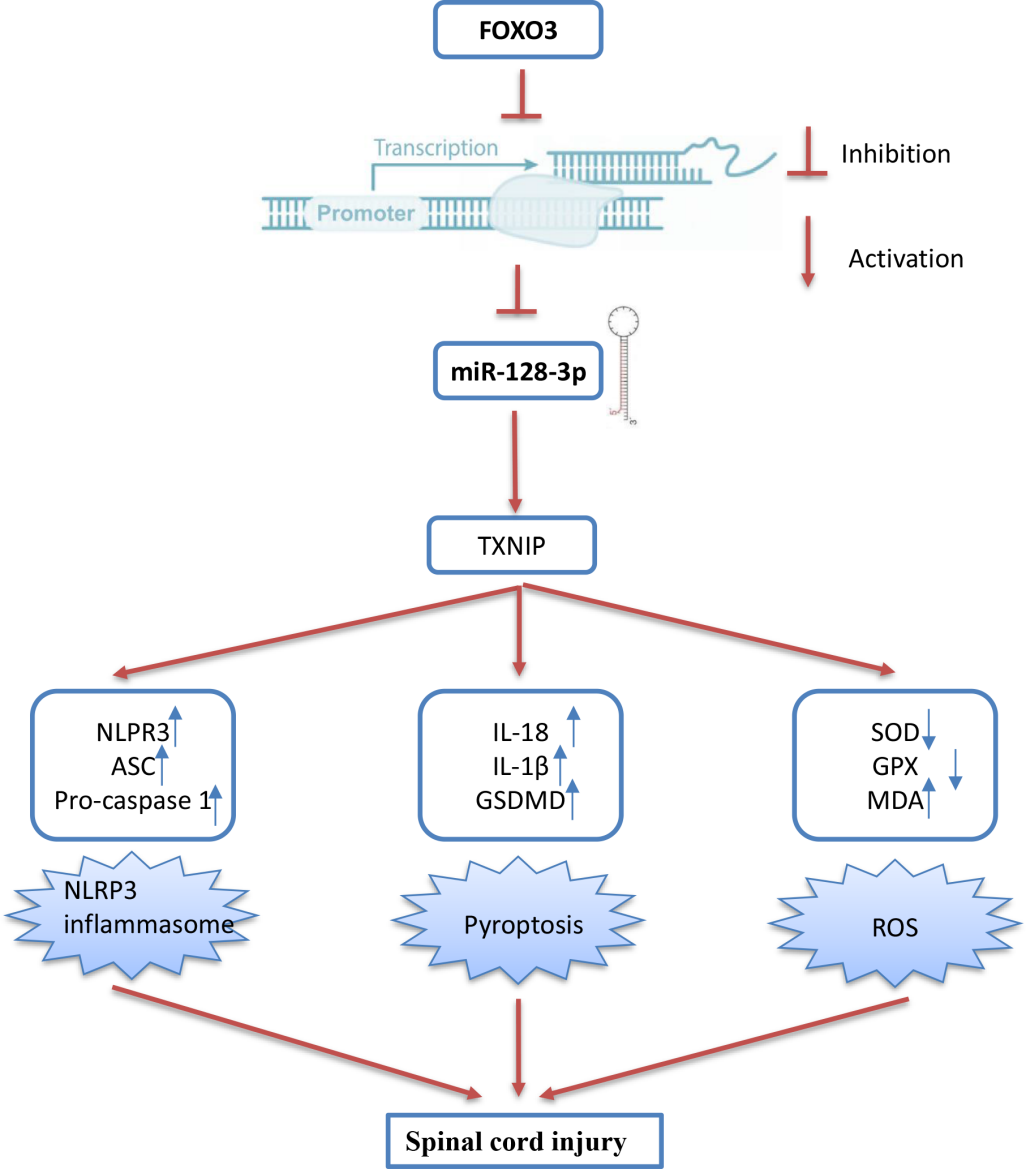
Schematic diagram of the present study. The downregulated miR-128-3p which is regulated by the transcription factor FOXO3, facilitates spinal cord injury progression in mice through promoting oxidative stress, cell pyroptosis and inflammation by targeting TNXIP.

Next, we further evaluated the effect of FOXO3 on oxidative stress, cell pyroptosis, and inflammation in the SCI cell model. As shown in **Figures 8E–H**, the levels of ROS and MDA were significantly increased, while the activity of SOD and GPX decreased in the LPS group compared with the control group. However, LPS-induced oxidative damage was alleviated by pcDNA-FOXO3 transfection. Moreover, the pyroptosis rate was significantly increased in LPS-treated BV2 cells, whereas the increased pyroptosis rate was markedly reduced by pcDNA-FOXO3 transfection. In addition, pyroptosis -induced inflammatory response (IL-1β, IL-18, and TNF-α) was also reversed by pcDNA-FOXO3 (**Figures 8I–L**). All these data indicated that FOXO3 overexpression has similar effects with miR-128-3p mimics *in vitro*. Collectively, our results suggest that the downregulated miR-128-3p facilitates spinal cord injury progression in mice through promoting oxidative stress, cell pyroptosis and inflammation by targeting TNXIP (**Figure 9**).

## Discussions

In the present study, miR-128-3p was notably decreased in the spinal cord tissues of SCI mice. Upregulation of miR-128-3p could improve functional behavioral recovery and alleviate pathological damage. Additionally, our research has revealed that upregulated ROS during secondary injury can trigger the activation of the NLRP3 inflammasome, leading to pyroptosis, and miR-218 upregulation can ameliorate this process. In SCI cell model, TNXIP was identified as a target of miR-128-3p and overexpression of TNXIP reversed the ameliorative effects of miR-128-3p on ROS and pyroptosis. Moreover, the expression of miR-128-3p was regulated by the transcription factor FOXO3, and overexpressing FOXO3 yielded similar functional outcomes to those observed with miR-128-3p overexpression. Our findings suggest that miR-128-3p may act as a promising therapeutic candidate for SCI

Accumulating evidence elucidates that dysregulated miRNAs are implicated in the pathogenesis of spinal cord injury. For example, miR-182-5p alleviated spinal cord injury by inhibiting inflammation and apoptosis by modulating the TLR4/NF-κB pathway (39). miR-299a-5p protected against spinal cord injury by activating the AMPK pathway (40). Over-expression of miR-223 was demonstrated to alleviate the spinal injury to some extent, decrease the inflammation, and improve nervous system function *in vivo* and *in vitro* (41). Several studies have focused on comprehensive investigations into the biological functions of miR-128-3p. miR-128-3p was significantly downregulated in the SCI rat model and overexpression of miR-128-3p protected neurons from neuroinflammation and apoptosis during spinal cord I/R injury partially by downregulating SP1 (42), which was consistent with our findings. In addition, a previous study has shown that down-regulation of miR-128-3p in murine microglial cells may contribute to the development of neuropathic pain following SCI via activation of P38 (24). We found miR-128-3p was significantly decreased in the mouse SCI model, indicating that miR-128-3p may be involved in the pathogenesis of SCI. Subsequently, we successfully created a SCI mouse model and agomiR-128-3p injection improved functional behavioral recovery, alleviated spinal cord edema, and relieved pathological injury, suggesting that targeting miR-128-3p may be a promising therapeutic target for SCI. However, the role and mechanism of miR-128-3p in the secondary injury after SCI was not reported.

SCI is a complex pathological process, including pathological injury of the spinal cord mediated by oxidative stress. It has been reported that when SCI occurs, the initial mechanical trauma of SCI can compromise blood flow to the affected area of the spinal cord, leading to ischemia and hypoxia, which can trigger the production of ROS within cells (13, 43). Consistently, we herein observed that SCI-induced ROS generation was reduced by agomiR-128-3p treatment, accompanied by increased SOD and GPX, and reduced MDA levels in the spinal cord. *In vitro*, we confirmed that miR-128-3p upregulation alleviated LPS induced oxidative stress, which verified the findings of the *in vivo* experiment.

Pyroptosis is called as a recently identified programmed cell death regulated by the activation of caspase-1, caspase-4/5/11 and GSDMD regulated signaling pathways and release of several inflammatory mediators such as IL-1β and IL-18 (44). Current studies have shown that ROS can activate the NLRP3 inflammasome in microglia, then lead to the pyroptotic death of microglia (45). NLRP3, the most widely studied inflammasome involved in many damage signaling pathways and inflammatory reactions, has been verified to be activated to induce nonbacterial inflammation by many nonbiological danger signals, such as ROS (46). The NLRP3 activation and ASC recruitment mediate the caspase-1 and regulate the maturation and secretion of proinflammatory factors, and NLRP3 inactivation dramatically protects against motor dysfunction and sensory hypersensitivity in SCI mice (13, 43). Chen et al. Found that WTAP participates in neuronal damage by protein translation of NLRP3 in an m6A-YTHDF1-dependent manner after traumatic brain injury (TBI) (47). Additionally, He et al. found that inhibiting NLRP3-induced pyroptosis could alleviate sepsis-induced brain injury (48). In this study, we observed that NLRP3, ASC, caspase-1, GSDMD, IL-1β, IL-18 and TNF-α protein expression levels were markedly increased after SCI, indicating that the NLRP3 inflammatory activation-mediated pyroptosis is an important pathological mechanism in SCI, which was consistent with a previous study in Ischemia/reperfusion (I/R) injury (49). We also demonstrated that agomiR-128-3p treatment improved SCI mice by suppressing pyroptosis cascade of NLRP3 inflammasome activation. *In vitro*, we confirmed that miR-128-3p upregulation alleviated LPS induced cell injury by stimulating a decrease of NLRP3 inflammasome activation-induced pyroptosis, which further verified the findings of the *in vivo* experiment.

TXNIP also known as TBP2, is known to interact with thioredoxin (Trx) and inhibit its antioxidant functions. When cells are in a quiescent state, TXNIP interacts with the redox domain of Trx and is considered a negative regulator of Trx. However, when intracellular ROS are increased, Trx is oxidized, thus leading to the dissociation of TXNIP from Trx, which subsequently interacts with NLRP3, leading to the assembly and activation of the NLRP3 inflammasome (50). In this study, we identified TXNIP as a direct target of miR-128-3p in regulating SCI. TXNIP is traditionally identified to participate in the pathogenesis of various human diseases such as kidney disease, neurodegeneration, and diabetes; however, emerging studies reveal that it also plays critical roles in regulating inflammation, oxidative stress, pyroptosis and tissue injury (51, 52). For example, TXNIP regulated the production of ROS through mitochondria and NADPH oxidase under high glucose conditions (53, 54). Consistently, we found that TXNIP overexpression evidently reversed the inhibitory effects of miR-128-3p on SCI-induced oxidative stress *in vitro* model. Additionally, TXNIP overexpression abolished the anti-pyroptosis and anti-inflammatory effect of baicalein on pancreatic acinar cells (PACs) by regulating NLRP3/Caspase-1 pathway (55). And results from Jia et al. demonstrated that Metformin protected against intestinal ischemia-reperfusion injury and cell pyroptosis via TXNIP-NLRP3-GSDMD pathway (56). In line with these findings, we also found that miR-128-3p upregulation significantly reduced TXNIP expression in SCI cell model, thereby reducing SCI-induced inflammation and pyroptosis.

Several studies have indicated that the transcription factor (TF)-miRNA signal is closely associated with organ injuries (57, 58). For example, Zhao et al. indicated that NF-κB(p65) promotion of miR-99b can aggravate ALI in mice by down-regulating the expression of PRDM1 (59). Li et al. showed that transcription factor Foxd3 induced spinal cord ischemia-reperfusion injury by potentiating miR-214-dependent inhibition of Kcnk2 (60). Therefore, TF induced dysregulation of miRNAs may be a significant cause of aberrant miRNA expression in SCI. In our study, TF regulating miR-128-3p promoters were predicted using the JTRANSFAC database, and FOXO3 was identified as a key upstream regulatory molecule. We also noted that miR-128-3p expression level was significantly enhanced by upregulation of FOXO3 expression. FOXO3, a member of the Forkhead box O (FOXO) family of transcription factors, plays a significant role in regulating inflammation, oxidative stress, and tissue injury (61). In SCI, decreased levels of FOXO3 are involved in axonal regeneration and the proliferation of glial cells (62). FOXO3 can influence the inflammatory response post-SCI by regulating the production of pro-inflammatory cytokines such as IL-6 and TNF-α (63). In this study, we further investigated the roles of FOXO3 in SCI cell model. The results showed that overexpression of FOXO3 suppressed LPS-induced oxidative stress, pyroptosis, and inflammation *in vitro*, which is similar to the effects of miR-128-3p upregulation. Taken together, our findings suggest that the FOXO3/miR-128-3p axis may contribute to the secondary injury process in SCI.

However, there were still some limitations in this study. For example, we have screened multiple differentially expressed miRNAs from GEO databases, thus the functions of the other miRNAs will be further investigated in the future. Similarly, TXNIP is not unique as a target gene of miR-128-3p, which may lead to other mechanisms besides NLRP3-mediated pyroptosis being involved in the damage process regulated by miR-128-3p. However, our preliminary findings verified the role of FOXO3/miR-128-3p/NLRP3-mediated pyroptosis axis in post-SCI neuroinflammation and subsequent neuropathology. Hence, further research is warranted to explore therapeutic strategies targeting miR-128-3p and NLRP3-mediated pyroptosis in neurons to reveal their potential therapeutic value for SCI.

## Conclusion

In summary, our findings provide important insights into the mechanisms through which miR-128-3p has a protective role in SCI. It targets TXNIP to regulate oxidative stress, inflammation and pyroptosis, thus alleviating secondary injury in SCI. In addition, we also verified that the aberrant expression of miR-128-3p is emphasized to be under the regulation of the transcription factor FOXO3. Therefore, FOXO3/miR-128-3p/NLRP3-mediated pyroptosis axis may serve as a new therapeutic target for the prevention and treatment of SCI.

## Data Availability

The original contributions presented in the study are included in the article/supplementary material. Further inquiries can be directed to the corresponding authors.
